# Body Awareness as a Protective Factor against Suicidal Orientations in College Students

**DOI:** 10.3390/bs14050358

**Published:** 2024-04-25

**Authors:** Olga Lucia Montoya-Hurtado, Renato Sobral-Monteiro-Junior, Cyndi Yacira Meneses-Castaño, Consuelo Sancho-Sánchez, Antonio Martínez-Sabater, Pilar Andrés-Olivera, Pilar Sanchez-Conde, Jesús Pérez Sánchez-Toledo, José María Criado-Gutiérrez, Laura Criado-Pérez, Juan Luis Sánchez-González, Raúl Juárez-Vela

**Affiliations:** 1 Doctoral Program in Program in Health, Disability, Dependency, and Well-Being, University of Salamanca, 37007 Salamanca, Spain; olga.montoya@ecr.edu.co; 2 Research Department, Escuela Colombiana de Rehabilitación, Bogotá 110121, Colombia; cyndi.meneses@ecr.edu.co; 3 Program de Pós-Graduação em Ciências da Saúde, Universidade Estadual de Montes Claros, Montes Claros 39406, Brazil; renato.monteiro@unimontes.br; 4 Department of Physiology and Pharmacology, Faculty of Medicine, University of Salamanca, 37007 Salamanca, Spain; sanchoc@usal.es (C.S.-S.); jmcriado@usal.es (J.M.C.-G.); 5 Nursing Care and Education Research Group (GRIECE), Nursing Department, Universität de Valencia, 46010 Valencia, Spain; antonio.martinez-sabater@uv.es; 6 Grupo Asociado de Investigación en Cuidados (INCLIVA), Hospital Clínico Universitario, 46010 Valencia, Spain; 7 Psychiatry Service, University of Salamanca Healthcare Complex (CAUSA), 37007 Salamanca, Spain; mpolivera@usal.es; 8 Psychiatric Unit, School of Medicine, University of Salamanca, 37007 Salamanca, Spain; 9 Department of Surgery, School of Medicine, University Health Care Complex of Salamanca, University of Salamanca, 37007 Salamanca, Spain; pconde@usal.es; 10 Institute of Biomedicine of Salamanca (IBSAL), Prevention, and Early Intervention in Mental Health (PRINT), 37007 Salamanca, Spain; jesusperez@usal.es (J.P.S.-T.); raul.juarez@unirioja.es (R.J.-V.); 11 Department of Nursing, Faculty of Health Sciences, University of La Rioja, 26004 Logroño, Spain; 12 Department of Nursing and Physiotherapy, Faculty of Nursing and Physiotherapy, University of Salamanca, 37007 Salamanca, Spain; juanluissanchez@usal.es

**Keywords:** body awareness, interoception, suicide prevention, attempted suicide

## Abstract

In this study, binary logistic regression and linear regression analyses were used to examine the relationship between interoceptive body awareness and suicidal orientation among Colombian university students. Additionally, the bootstrap technique was employed to resample and estimate the distribution of the data. The results support the idea that greater interoceptive awareness may protect against suicidal orientation by improving emotional regulation. An inverse relationship was found between interoceptive awareness and suicidal ideation. These findings align with previous literature emphasizing the importance of body awareness for emotional well-being. Further longitudinal research is needed to explore this relationship more deeply.

## 1. Introduction

Suicide poses a complex challenge to public health and is influenced by various biological, psychological, social, and environmental factors. According to the three-stage theory, which includes suicidal ideation, planning, and execution, identifying warning signs in each phase is crucial for prevention and intervention [1]. The World Health Organization (WHO) recognizes suicide as the fourth leading cause of death among individuals aged 15–19 years in the Americas [2]. In Colombia, young adults between 20 and 24 years of age, and especially those aged 18 and 19 years, have a high suicide rate. Therefore, understanding the protective factors is essential for effective prevention and treatment, particularly among university students [3].

On the other hand, interoception, defined as the ability to perceive and recognize internal body signals such as pain, temperature, hunger, and thirst, plays a vital role in the regulation of physical and emotional well-being. This ability differs from external perception, which processes environmental stimuli and is responsible for monitoring and regulating internal bodily functions to maintain homeostatic balance. Fundamental to understanding the internal physiological state, interoception involves both the perception of basic bodily sensations and the management of autonomic motor control, thus distinguishing it from the exteroceptive system, which influences somatic motor activity such as cutaneous perception and proprioception. This ability is crucial not only for the regulation of internal bodily functions but also for the adaptation of the organism to changes in its physiological state, thus ensuring well-being and survival [4].

In addition, interoception plays an essential role in the generation of emotions and in the development of body awareness and perception of the “self” as an emotional and conscious entity. Its importance extends beyond the physical realm as it is linked to various psychological conditions. Alterations in this capacity can affect emotional regulation and the ability to appropriately interpret and respond to internal cues, which is associated with anxiety, eating, and mood disorders. Therefore, a thorough understanding of interoception is critical to address these mental health issues [5].

Individuals with suicidal tendencies may have a reduced interoceptive sensitivity. Those who have attempted suicide have been observed to have reduced interoceptive sensitivity compared with those who have only contemplated or planned suicide. Furthermore, impaired interoceptive sensitivity is indirectly related to suicide attempts through mediating variables. It is proposed that individuals with suicidal ideation do not necessarily lack the ability to recognize their own bodily cues but may feel less able to use these cues beneficially. Differences between individuals with and without suicidal ideation are often influenced primarily by depressive symptoms [6].

Interoceptive dysfunction, which involves disconnection from these bodily sensations, is associated with several psychiatric disorders and may be a modifiable risk factor for suicidal ideation and behavior. This disconnection facilitates self-harm, because it may be easier to harm a body to which one does not feel emotionally connected. Interoceptive dysfunction may not only differentiate individuals with suicidal ideation from those who actually attempt suicide but may also be a modifiable risk factor [7].

The importance of interoception in mental health is highlighted by discussions on how individuals who are more attuned to bodily responses experience emotions with greater intensity. It is also emphasized that interoception is relevant to emotional theories that propose a basis for emotional states in the central representation and perception of changes in bodily physiology. These aspects underscore the link between interoception and emotional experience, which may be relevant in the context of mental health and emotional well-being [8].

In Colombia, mental health care in the university setting is a priority for both the Ministry of Health and Ministry of Education, as reflected in the Guidelines on Mental Health and Psychosocial Disability in the Colombian Higher Education System. These guidelines emphasize the importance of implementing policies and practices aimed at fostering a supportive and emotional caring environment within higher education institutions. This includes not only the availability of counseling and psychological support services but also the active promotion of healthy self-care and wellness practices among students [9].

In this context, body awareness, particularly interoceptive body awareness, emerges as an important but under-explored factor [8]. Despite increasing attention paid to mental health in the university setting, research addressing the conceptual and methodological challenges associated with health promotion is still lacking.

Therefore, this study aimed to investigate whether there is a predictive relationship between interoceptive awareness and suicidal intent by examining the ability of the variables to predict the latter. In addition, we explored how one variable might affect the other and used methods such as bootstrapping to obtain more precise estimates of results when the data deviated from a normal distribution.

## 2. Materials and Methods

### 2.1. Study Type

A cross-sectional observational study was conducted to examine the relationship between body image and suicidal orientation among rehabilitation science students at a Colombian university in 2023.

### 2.2. Population and Sample

Convenience sampling was employed to recruit undergraduate rehabilitation science students from a Colombian university. This method was chosen because of the accessibility and availability of the target population, which facilitated efficient data collection. Given the study’s focus on the specific demographics of university students, convenience sampling enabled a quick and practical participant selection process. The selection criteria were focused on undergraduate rehabilitation science students at the Department of Colombian University, ensuring homogeneity in academic and disciplinary contexts to enhance the comparability of results within the sample. The bootstrapping method was utilized to increase the sample size and enhance the precision of the results, particularly for non-normally distributed data. This technique involves generating multiple samples from the original data by sampling with replacement, allowing for a more accurate parameter estimation and robust assessment of statistical significance. Online questionnaires were distributed via Google Forms in three sessions with a researcher available to address any queries or concerns.

### 2.3. Instruments

The instruments used in this study were the Multidimensional Assessment of Interoceptive Awareness (MAIA) and the Inventory of Suicide Orientation (ISO-30). These instruments were validated and applied to Colombian university students.

Multidimensional Assessment of Interoceptive Awareness (MAIA): The MAIA is a questionnaire used to measure interoceptive body awareness in individuals. It consists of 32 items categorized into eight dimensions, evaluating interoceptive awareness using a Likert scale ranging from 0 (never) to 5 (always). It provides a total score for the level of body awareness and dimensional assessment. The MAIA has been found to have high reliability, with a Cronbach’s alpha of 0.90 and an omega coefficient of 0.96 in a sample of Colombian university students. Each dimension of the MAIA is defined based on specific aspects of body awareness, such as attention to interoceptive signals, emotional regulation, and confidence in interpreting bodily signals [10,11].

Inventory of Suicide Orientation (ISO-30): The ISO-30 is a questionnaire comprising 30 questions designed to detect the possible presence of suicidal orientation. The questions are formulated as positive and negative, with responses given on a four-point Likert scale (0 = disagree, 1 = partially disagree, 2 = partially agree, and 3 = strongly agree). The ISO-30 assesses the presence and intensity of suicidal orientation over a 30-day period. It has been found to have good reliability, with a Cronbach’s alpha of 0.899 in a population of Colombian university students. This questionnaire provides relevant information for the early identification of individuals at risk of suicidal behaviors and for appropriate intervention and treatment [12].

### 2.4. Statistical Analysis

Statistical analysis of interoceptive body awareness and suicidal orientations in university students was conducted using JASP software 0.13.1. This included the following:

- MAIA scoring: Based on a median of 2.9, where higher scores indicate better interoceptive awareness, and lower scores indicate poorer awareness. The samples were divided into quartiles of 25, 50, and 75%.
- ISO-30 scoring: Based on a median score of 34, where scores equal to or above this indicate higher suicidal intent and scores below indicate lower intent. The sample was similarly divided into quartiles of 25%, 50%, and 75%.
- Comparison of MAIA and ISO-30 variable frequencies between males and females: This helped to examine potential gender differences in body awareness and suicidal intent.
- Chi-squared test: This was used to examine differences in variable frequencies between males and females, providing additional information on how these gender differences may affect the relationship between body awareness and suicidal orientation.
- Binary logistic regression: This study assessed the association between MAIA and ISO-30 scores, allowing determination of whether a significant relationship exists between body awareness and suicidal intent after controlling for other relevant factors.
- Evaluation of area under the curve, sensitivity, and specificity: Information on the accuracy of the statistical models used and their ability to predict suicidal intent based on body awareness.
- Linear regression: To investigate whether the total MAIA score explains the variation in the total ISO-30 score, aiding a better understanding of the relationship between body awareness and suicidal intent in quantitative terms.

## 3. Results

Based on the division of the sample according to the median cut-off points for the MAIA and ISO-30, it was observed that a higher percentage (51.8%) of students fell below the median in the MAIA percentile, indicating lower interoceptive awareness in this group. This categorization approach enabled the identification of individuals with lower interoceptive body awareness. Additionally, those who scored above the median on the ISO-30 were categorized as having higher interoceptive awareness. These findings align with the data presented in Table 1.

The data, as shown in Table 2, illustrate the frequency distribution of participants by gender and their classification into MAIA and ISSO-30 percentiles. However, chi-squared tests and log odds ratio analyses demonstrate no significant association between gender and MAIA and ISSO-30 percentiles. This suggests that gender is not significantly related to interoceptive awareness and suicidal intent in this sample.

The logistic regression model presented in Table 3 indicates that between 16% and 21% of the variance in the ISSO-30 percentile is accounted for by the MAIA percentile (Cox and Snell R² = 0.16; Nagelkerke R² = 0.21; *p* < 0.01). Age does not exhibit a statistically significant influence in this model (considered as a confounding variable; *p* = 0.46). Furthermore, individuals with a MAIA score of 2.9 or higher demonstrate a significantly reduced likelihood of suicidal ideation, with an odds ratio of 0.17 (*p* = 2.41 × 10^−7^), representing a protective factor of 83%.

The performance metrics, as depicted in Table 4, highlight the model’s effectiveness in predicting suicidal intent based on the ISO-30 percentile. The AUC (area under the curve) value of 0.69 suggests a moderate discriminative power of the model. Sensitivity, which denotes the proportion of true positives correctly identified, stands at 0.71, indicating that the model accurately identifies suicidal individuals 71% of the time. Specificity, representing the proportion of true negatives correctly identified, is 0.70, indicating a 70% accuracy in identifying non-suicidal individuals. Accuracy, also referred to as positive predictive value, is 0.71, indicating the proportion of true positive predictions out of all positive predictions made by the model. Overall, these metrics suggest moderate performance in predicting suicidal intent.

The data from the Model Summary—ISO30_total_score indicate that the linear regression model applied to the ISO-30 total score yields a coefficient of determination (R²) of 31% in its fitted form, implying that the model explains approximately 31% of the variability in the ISO-30 total score. This suggests a moderate ability of the model to predict the ISO-30 total score.

These data represent an Analysis of Variance (ANOVA) conducted on the regression model. The table illustrates that the regression is statistically significant, with an F-value of 74.06 and an extremely low *p*-value (5.37 × 10^−15^), indicating the significance of the regression model. This suggests that at least one of the independent variables incorporated in the model significantly impacts the dependent variable (ISO30_total_score).

**Figure 1:**
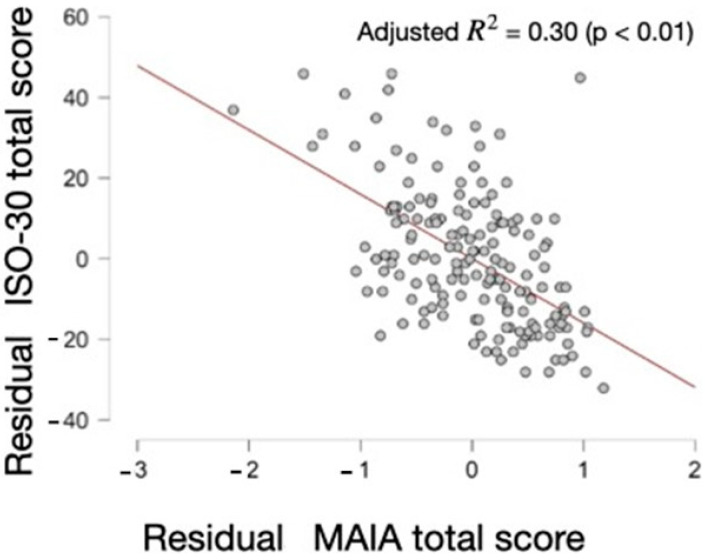
ISO-30 total score vs. MAIA total score.

In this analysis (Table 5, Table 6 and Table 7 and Figure 1), an inverse relationship between the MAIA score and ISO-30 is evident. As the MAIA score increases, the ISO-30 score decreases (B unstandardized = −15.95; *p* = 5.37 × 10^−15^ --> *p* < 0.000000000000005). Moreover, it was confirmed that there is no multicollinearity, as the tolerance values exceed 0.25 and the variance inflation factors (VIFs) are less than 4. By replicating the sample 1000 times (169,000 data points), it was observed that this relationship persists (B unstandardized = −16) and the confidence interval remains significant (−19.57, −11.60). This underscores the consistency and robustness of the relationship between the MAIA and ISO-30 scores.

The data presented illustrate the coefficients and collinearity diagnostics for the H_1_ model. According to the coefficients, there exists a significant negative relationship between the MAIA total score and ISO-30 total score (B = −16.00, *p* < 0.000000000000005), indicating that as interoceptive awareness increases, suicidality decreases. Furthermore, collinearity diagnostics reveal no significant collinearity issues between the variables in the model, enhancing the reliability of the obtained results.

## 4. Discussion

In our study, logistic regression modeling revealed that 16–21% of the variability in the ISO-30 percentile could be accounted for by the MAIA percentile, suggesting a potential role of interoceptive awareness in predicting suicidal orientations. This finding was further supported by the model performance analysis, demonstrating a moderate ability to predict suicidal tendencies based on the ISO-30 percentile. Additionally, linear regression analysis indicated a significant negative association between the MAIA and ISO-30 scores, implying that higher interoceptive awareness is linked to lower levels of suicidal ideation. This relationship remains consistent across multiple samples, indicating reliability.

Similar studies underscored the importance of addressing interoceptive body awareness. For instance, a study involving 319 adults undergoing specialized treatment for eating disorders found that low body confidence was associated with increased severity of suicidal ideation [13]. Research on non-suicidal self-injury (NSSI) has explored how difficulties in body perception could relate to this behavior, suggesting potential areas for clinical intervention [14,15,16]. Furthermore, investigations into the relationship between interoceptive dysfunction and suicidal behavior in participants with a history of suicide at-tempts have emphasized the importance of further exploring the association between in-ternal body perception and mental health [17,18].

Additionally, a study assessed the impact of a Biological Movement (BM) program, based on mindful movement, on the psychological well-being and interoceptive awareness of participants. The study implemented an 8-week training program for kinesiology students at the University of Perugia, Italy. The results indicated significant improvements in interoceptive awareness and positive mental health among the participants. The BM program enhanced participants’ psychological well-being and fostered a stronger connection between physical and emotional sensations [19].

Our study supports previous research highlighting the importance of body aware-ness in mental health and emotional well-being, suggesting that a greater ability to perceive and understand internal bodily sensations may offer protection against suicidal orientation. Interoception concepts, including interoceptive accuracy and sensitivity, can help us understand how we experience and relate to our internal bodily sensations.

Interoception, the awareness of internal bodily sensations, could benefit from techniques such as mindfulness and meditation, thereby strengthening the mind–body connection. These practices help individuals be more present in their bodies, recognize and regulate their physiological responses, and better understand their emotional states. Mindfulness enhances internal awareness, which can reduce suicide risk by promoting greater acceptance of internal experiences and improving emotional regulation. In summary, mindfulness strengthens interoception and reduces suicide risk by enhancing emotional awareness and managing distress [8,20].

One study examined suicide risk among college students and how alexithymia, or difficulty verbally identifying and expressing emotions, mediates the relationship between mindfulness and suicide risk. Approximately 13.5% of participants were found to be at risk for nonclinical suicide, a lower rate than that in previous studies, possibly due to the low level of stress among first-year students. Females showed a higher likelihood of suicide risk, possibly due to higher psychological stress and difficulty in identifying feelings. The results indicated that mindfulness was negatively related to alexithymia and suicide risk and that alexithymia mediated the relationship between mindfulness and suicide risk, especially in females. This suggests that mindfulness may promote greater emotional awareness and reduce suicide risk by improving emotion regulation. In addition, increases in mindfulness were associated with decreases in difficulty identifying emotions, which may be related to changes in insular function and structure. This study has some limitations, such as the lack of generalizability to other populations and the inability to establish causal relationships due to its cross-sectional design [21]. 

A cross-sectional observational study recruited 537 individuals via Amazon’s Mechanical Turk (MTurk), aged between 18 and 71 years. The Multidimensional Assessment of Interoceptive Awareness and a series of questions regarding the presence of lifetime suicidal ideation, plans, and attempts were used. The results indicate that interoception deficits could play a significant role in predicting and treating suicidality. Those with a history of suicidal thoughts exhibited greater concern or emotional distress towards negative bodily sensations associated with difficulties in emotional regulation and anxiety. Conversely, individuals who have attempted suicide tend to ignore or distract themselves from uncomfortable bodily sensations, possibly engaging in activities that increase their capability to commit suicide. Lack of self-regulation through attention to bodily sensations may be linked to overwhelming levels of arousal and distress, contributing to suicide attempts as a means of alleviating negative emotions. Finally, those with a history of suicidality have less trust in their bodily sensations, which may facilitate self-destructive behavior [22].

Our study supports the use of strategies that promote interoceptive body awareness as a complement to other interventions in primary healthcare, as demonstrated by a single-group clinical trial involving 43 adolescents with depression in primary care, predominantly of Hispanic/Latino origin and female sex. Self-report measures were assessed using the Childhood Depression Inventory-2, the Suicidal Ideation Questionnaire, the Mindfulness Scale, Self-Efficacy for Depressed Adolescents, the rumination subscale of the Children’s Response Styles Questionnaire, and an acceptability questionnaire. A 10-week Mind–Body Skills Group program was conducted in primary care. The participants completed assessments at three time points: at baseline, post-intervention, and at the 3-month follow-up. We observed a significant improvement in total depression scores post-intervention, as well as improvements in mindfulness, self-efficacy, rumination, and suicidal ideation [23].

Assessing interoceptive body awareness can support primary care strategies. Regarding interoception, the ability to sense internal bodily sensations is compromised in individuals with suicidal tendencies. Two studies compared interoception among individuals with varying degrees of suicidal tendencies and found that those with suicidal tendencies had poorer interoception than the controls. In addition, those who had a recent suicide attempt had greater interoceptive deficits than those who had not. These findings suggest that impaired interoception may be significant for engaging in serious self-harm and that improving the connection with the body could help prevent suicidal behavior [24].

Primary healthcare can assist in detecting early warning signs of mental disorders and in timely referral to specialized mental health services when necessary. Interdisciplinary collaboration between primary healthcare personnel and mental health professionals can significantly enhance the quality and effectiveness of mental health services in university settings. Furthermore, to strengthen interoceptive body awareness in the context of university primary healthcare, it is essential to implement specific strategies aimed at enhancing students’ mind–body connections. These strategies may include integrating mindfulness and mindful awareness practices, promoting mindful physical activities, educating about the importance of body awareness in mental health, and incorporating relaxation techniques and emotional self-regulation [25,26,27,28,29].

## 5. Conclusions

Our study revealed a significant association between interoceptive awareness and suicidal orientation in Colombian university students. Through logistic regression analysis, we underscored this correlation, emphasizing the role of interoceptive awareness in predicting suicidal orientation. This was further supported by an inverse correlation between the MAIA and ISO-30 scores, suggesting that higher interoceptive awareness correlates with lower suicidal orientation. These findings are consistent with the existing literature, which highlights the importance of body awareness in mental health and emotional well-being.

It is important to acknowledge that our study focuses on correlational relationships without delving into the underlying causal mechanisms. The cross-sectional nature of the study design prevents us from establishing definitive causal relationships, and the limitation of the sample to Colombian university students may affect the generalizability of the findings.

To address these gaps, future research should focus on exploring the causal mechanisms, employing longitudinal designs, and considering more diverse populations. This would enhance our understanding of the applicability of the results to clinical practice and preventive interventions. Further investigation is necessary to fully understand these relationships.

There is an evident need to conduct longitudinal assessment and intervention studies in this population to deepen our understanding of these phenomena and develop effective prevention and care strategies. Longitudinal studies would allow us to follow students over time, providing information on how the relationship between interoceptive awareness and suicidal orientation changes. This would help identify potential risk and protective factors as well as allow for better understanding of the underlying mechanisms linking interoceptive awareness to mental health and suicidal behavior.

Longitudinal intervention studies could evaluate the effectiveness of different interventions aimed at improving interoceptive awareness and reducing suicidal orientation in university students. These interventions could include mindfulness programs, cognitive-behavioral therapy, mindful physical activities, and other approaches focused on strengthening the mind–body connection.

## Figures and Tables

**Table 1:** Descriptive statistics.

**Gender**					
	**MAIA Percentile**		**ISO-30 Percentile**		
	**Female**	**Male**	**Female**	**Male**	
Valid	135	34	135	34	
Missing	0	0	0	0	
**Frequencies for MAIA Percentile**					
**Gender**	**MAIA Percentile**	**Frequency**	**Percent**	**Valid Percent**	**Cumulative Percent**
Female	Below median 2.90	70	51.85	51.85	51.85
	At or above median 2.90	65	48.15	48.15	100.00
	Missing	0	0.00		
	Total	135	100.00		
Male	Below median 2.90	15	44.12	44.12	44.12
	At or above 2.90	19	55.88	55.88	100.00
	Missing	0	0.00		
	Total	34	100.00		
**Frequencies for ISSO-30 Percentile**					
**Gender**	**ISSO-30 percentile**	**Frequency**	**Percent**	**Valid Percent**	**Cumulative Percent**
Female	Below median 34	65	48.15	48.15	48.15
	At or above median 34	70	51.85	51.85	100.00
	Missing	0	0.00		
	Total	135	100.00		
Male	Below median 34	19	55.88	55.88	55.88
	At or above median 34	15	44.12	44.12	100.00
	Missing	0	0.00		
	Total	34	100.00		

**Table 2:** Contingency tables.

**Contingency MAIA**				
	**Gender**			
**MAIA Percentile**	**Female**	**Male**	**Total**	
Below median 2.90	70	15	85	
At or above median 2.90	65	19	84	
Total	135	34	169	
**Chi-Squared Tests**				
	**Value**	**df**	***p***	
Χ²	0.65	1	0.42	
Χ² continuity correction	0.38	1	0.54	
N	169			
**Log Odds Ratio**				
		**95% Confidence Intervals**		
	**Log Odds Ratio**	**Lower**	**Upper**	***p***
Odds ratio	0.31	−0.45	1.07	
Fisher’s exact test	0.31	−0.51	1.14	0.45
**Contingency ISO-30**				
	**Gender**			
**ISSO-30 Percentile**	**Female**	**Male**	**Total**	
Below median 34	65	19	84	
At or above median 34	70	15	85	
Total	135	34	169	
**Chi-Squared Tests**				
	**Value**	**df**	***p***	
Χ²	0.65	1	0.42	
Χ² continuity correction	0.38	1	0.54	
N	169			
**Log Odds Ratio**				
		**95% Confidence Intervals**		
	**Log Odds Ratio**	**Lower**	**Upper**	***p***
Odds ratio	−0.31	−1.07	0.45	
Fisher’s exact test	−0.31	−1.14	0.51	0.45

**Table 3:** Model summary: ISSO-30 percentile.

**Model**	**Deviance**	**AIC**	**BIC**	**df**	**Χ²**	***p***	**McFadden R²**	**Nagelkerke R²**	**Tjur R²**	**Cox and Snell R²**
H_0_	234.28	236.28	239.41	168						
H_1_	204.73	210.73	220.12	166	29.55	3.83 × 10^−7^	0.13	0.21	0.17	0.16
**Coefficients**										
					**Wald Test**			**95% Confidence Interval**		
								**(Odds Ratio Scale)**		
	**Estimate**	**Standard Error**	**Odds Ratio**	**z**	**Wald Statistic**	**df**	***p***	**Lower Bound**	**Upper Bound**	
(Intercept)	1.40	0.77	4.08	1.83	3.36	1	0.07	0.91	18.29	
Age	−0.02	0.03	0.98	−0.73	0.53	1	0.46	0.91	1.04	
MAIA percentile (At or above median 2.90)	−1.75	0.34	0.17	−5.16	26.67	1	2.41 × 10^−7^	0.09	0.34	

Note: ISSO-30 percentile level “At or above median 34” coded as class 1.

**Table 4:** Evaluation of model performance.

**Performance Metrics**						
		Value				
AUC		0.69				
Sensitivity		0.71				
Specificity		0.70				
Precision		0.71				
**Model Summary—ISO30_total_score**						
**Model**	**R**	**R²**	**Adjusted R²**	**RMSE**		
H_0_	0.00	0.00	0.00	17.00		
H_1_	0.55	0.31	0.30	14.19		
**ANOVA**						
**Model**		**Sum of Squares**	**df**	**Mean Square**	**F**	***p***
H_1_	Regression	14,920.36	1	14920.36	74.06	5.37 × 10^−15^
	Residual	33,643.51	167	201.46		
	Total	48,563.87	168			

Note: The intercept model has been omitted, as it does not provide meaningful information.

**Table 5:** Coefficients.

							95% CI		Collinearity Statistics	
Model		Unstandardized	Standard Error	Standardized	t	*p*	Lower	Upper	Tolerance	VIF
H_0_	(Intercept)	35.14	1.31		26.87	1.05 × 10^−62^	32.55	37.72		
H_1_	(Intercept)	80.47	5.38		14.96	1.21 × 10^−32^	69.85	91.09		
	MAIA total score	−15.95	1.85	−0.55	−8.61	5.37 × 10^−15^	−19.61	−12.29	1.00	1.00

**Table 6:** Bootstrap coefficients.

Model		Unstandardized	Bias	Standard Error	95% bca * CI	
					Lower	Upper
H_0_	(Intercept)	35.13	−0.05	1.27	32.57	37.61
H_1_	(Intercept)	80.40	−0.20	5.89	68.08	91.80
	MAIA total score	−16.00	0.07	2.00	−19.57	−11.60

* Bias-corrected and accelerated. Note: Bootstrapping based on 1000 replicates. Note: Coefficient estimate is based on the median of the bootstrap distribution.

**Table 7:** Collinearity diagnostics.

Model	Dimension	Eigenvalue	Condition Index	Variance Proportions	
				(Intercept)	MAIA Total Score
H_1_	1	1.98	1.00	0.01	0.01
	2	0.02	9.75	0.99	0.99

Note: The intercept model is omitted, as no meaningful information can be shown.

## Data Availability

Data are available on request from the first author.
